# Health Concerns of Adolescents and Adults With Spina Bifida

**DOI:** 10.3389/fneur.2021.745814

**Published:** 2021-11-12

**Authors:** Jessica Starowicz, Caitlin Cassidy, Laura Brunton

**Affiliations:** ^1^Faculty of Health Sciences, Health and Rehabilitation Sciences, Western University, London, ON, Canada; ^2^Schulich School of Medicine and Dentistry, Physical Medicine and Rehabilitation and Paediatrics, Western University, London, ON, Canada; ^3^Faculty of Health Sciences, School of Physical Therapy, Western University, London, ON, Canada

**Keywords:** spina bifida (SB), health concerns, healthcare transition, adolescents, adults, multidisciplinary health care, care coordination, medical home

## Abstract

Due to advancements in medical care, people with spina bifida (SB) are surviving well into adulthood, resulting in a growing number of patients transitioning to an adult sector unequipped to care for people with chronic rehabilitative and medical needs. The Transitional and Lifelong Care (TLC) program is a multidisciplinary clinical service that compensates for this gap, providing comprehensive, coordinated care to adolescents, and adults with SB. As a relatively new clinical service, objective data about the patients using the service and their needs is scant. This study sought to identify the most common health concerns among TLC patients with SB at initial clinical consultation. A retrospective chart review of 94 patient charts was performed. Following data extraction, descriptive analyses were completed. The mean age of the sample was 29.04 ± 13.8 years. One hundred individual concerns and 18 concern categories were identified. On average, patients or care providers identified nine health concerns across various spheres of care, with care coordination being the most prevalent concern identified (86%). Patients also commonly had concerns regarding neurogenic bladder (70%), medications (66%), assistive devices (48%), and neurogenic bowel (42%). The numerous and wide-ranging health concerns identified support the need for individualised, coordinated care and a “medical home” for all adolescents and adults with SB during and following the transition to adult care. Health care providers caring for this population should continue to address well-documented health concerns and also consider raising discussion around topics such as sexual health, mental health, and bone health. Further research is required to understand how best to address the complex medical issues faced by adults with SB to maximise health and quality of life and improve access to healthcare.

## Introduction

Spina bifida (SB) is one of the most common permanently disabling conditions in humans (1). It is a non-progressive defect that results from incomplete closure of the neural tube during the first few weeks of embryonic development (1, 2). The most common form of SB is myelomeningocele, in which meninges and neural elements protrude through the spinal defect, necessitating neonatal surgical repair (3, 4). The clinical spectrum of SB ranges from mild to severe neurological impairment, with severity determined by several factors, including the size and location of the spinal cord malformation, the degree of spinal cord or nerve involvement in the defect, and whether the opening in the spine is covered (3, 4). The clinical consequences of SB are largely related to the degree of nervous system abnormalities resulting from the neural tube defect (4). Brain abnormalities, such as hydrocephalus and Chiari type II malformation, may occur and can be associated with learning disabilities, executive dysfunction, strabismus, precocious puberty, and/or epilepsy (4). The spinal cord malformation itself leads to a spectrum of sensory and motor impairments below the lesion level (typically located in the thoracic, lumbar, or sacral spine) and often to the development of neurogenic bowel and bladder dysfunction (4). The bony vertebral anomalies may lead directly to musculoskeletal consequences, including scoliosis and kyphosis (4, 5). Managing SB involves surgical closure of the defect, commonly initiated shortly after birth (though recent advances have resulted in more frequent consideration of pre-natal foetal surgery to repair the defect ((6))), and medical intervention throughout the lifespan (1). This may include continuous specialty care from urology, neurosurgery, orthopaedics, and physical medicine and rehabilitation, as well as access to therapy services from psychology and assistive technology services (4, 7).

Although life expectancy for people with SB has dramatically increased in the last several decades, the population is at increased risk for excess morbidity and early mortality in adulthood (1, 5, 8, 9). Adults with SB experience complex, multi-system disorders from childhood and adolescence throughout their lifespan (5, 8), co-morbid conditions and medical complexities that stem directly from their SB, as well as complications that may be indirectly related to SB, such as issues with infertility, poor mental health, obesity, and skin breakdown (5, 8). Moreover, adults with SB are at risk for health concerns and chronic health conditions common in the general population, such as hypertension, diabetes, and cancer (5). The consequences of SB may also impact cognition, such that specific learning disabilities or cognitive challenges that persist from childhood may impact independence and educational, social, and employment opportunities in adulthood (5, 8). As such, a similar level of health care service and comprehensive support must exist throughout adulthood as in childhood and adolescence (8). However, this is often not the case, and unsurprisingly, many adults with SB begin to experience a deterioration in their health soon after their discharge from paediatric health care services (8, 10, 11).

In Canada and internationally, youth with SB typically receive specialised, multidisciplinary health care services, often in well-structured paediatric rehabilitation centres (1, 12, 13). In these programs, children and adolescents with SB receive assessment, diagnosis, and management for their condition from a team of diverse health care practitioners, most often including orthopaedics, urology, neurosurgery, nursing, social work, as well as occupational, speech, and physical therapy (1, 4, 13). The approach to care is family-centred, comprehensive, and holistic, considering the physical, emotional, social, and behavioural aspects of development (14). However, transfer to adult care is inevitable and typically occurs between 18 and 21 years of age once adolescents become ineligible for paediatric services (12, 15). Unfortunately, many people with SB struggle to find similarly equipped adult health care programs that can effectively address their complex needs after discharge from their children's treatment centre (16).

A large body of scientific literature documents the many barriers inherent in the adult health care system—in Canada and internationally—that prevent successful transition and pose challenges to ongoing care (15, 17, 18). Although the usual adult model of care may be appropriate for the general population, transition to adult care can be particularly difficult for those with complex disabilities who face a lack of multidisciplinary care; fragmentation of services (separate appointments with different specialists across multiple settings must be booked and coordinated); scarce resources; poor coordination and communication between the adult and paediatric health care sectors and within the adult health care sector; lack of preparation or information for transition; and a paucity of health care professionals that are knowledgeable, properly trained, or interested in caring for adults with SB (15, 18). These barriers can pose further challenges for those people with SB facing more significant cognitive or psychosocial challenges, or those who are heavily reliant on caregivers (16). Difficulties in transition may also be exacerbated by significant cultural differences between paediatric and adult care services (12, 19, 20). Paediatric care is developmentally focused, multidisciplinary, and family-centred, where adult care tends to place a greater focus on independence, maturity, and single discipline visits (12, 19–21). Therefore, it is perhaps unsurprising that many adolescents and adults with SB and their families associate the transition experience with negative feelings (22). Several studies have also identified negative consequences of transitioning to adult care, including difficulty accessing health care services (8), failure to seek medical attention or access age-appropriate services (15), challenges accessing funding or insurance (8), higher use of emergency and inpatient health care (5, 8, 23), and a decline in health status over time (11).

As a first step in improving health care services for adults with SB, more must be known about the population and their health care needs. The Institute for Health Improvement's (IHI) Triple Aim Framework suggests that to achieve appropriate reform of the health care system, there is a need to: (1) improve the experience of care: (2) improve the health of populations; and (3) reduce per capita costs of health care, where the accomplishment of the aims requires a focus on a defined population and an organisation or an “integrator” that can coordinate health care services (24). Therefore, the purpose of the present study was to identify the most common health care concerns affecting patients with SB at the time of presentation to a transitional and lifelong care clinic. It was hypothesised that patients would present to the clinic with multiple health concerns spanning multiple clinical areas, and that the most frequently identified patient concerns would relate to common conditions specific to SB, such as neurogenic bowel and bladder, as well as common secondary consequences of SB, such as osteoporosis and pressure ulcers.

## Materials and Methods

### Study Purpose

The objectives of this study were to identify the clinical health care concerns among patients with SB presenting to the Transitional and Lifelong Care (TLC) program, located in London, Ontario, at the Parkwood Institute site of St. Joseph's Health Care.

### Study Design

This study used an observational design involving a retrospective medical chart review of initial TLC patient consultation encounters between 2014 (time of the program's inception) and December 2017. All patients who presented to the clinic during this time with a diagnosis of SB were included in the study. There were no restrictions on age (or other patient characteristics) as the TLC clinic is a lifelong care program where a significant proportion of people in adult care have unmet health needs. Ethics approval was initiated through the Lawson Health Research Institute and approved by the Health Sciences Research Ethics Board at Western University.

The TLC program was developed in line with best-practise recommendations around transition to adult care (25) and addresses the diverse needs of adolescents and adults with chronic childhood-onset disabilities, including SB. It functions both as a transition service and an ongoing clinical program (otherwise known as a “medical home”) that provides scheduled and as-needed support for patients with SB throughout their lifespan, with access to knowledgeable clinicians and coordination of services in one comprehensive care setting. The program houses a multidisciplinary team of health care practitioners, including a physiatrist, nurse practitioner, social worker, physiotherapist, occupational therapist, speech-language pathologist, registered dietitian, and rehabilitation assistant. In line with paediatric services offered to children with complex health care needs, this diverse team provides a holistic approach to health care, considering physical and psychosocial well-being. See Table 1 for specific program services.

**Table 1:** TLC services offered in initial consultation and follow-up visits.

**Initial consultation**	**Follow-up visits**
Transitional (“overlap”) clinics at Thames Valley Children's Centre for adolescents nearing transition Access to interdisciplinary services, including (but not limited to) physiotherapy, occupational therapy, speech and language therapy assessment and treatment, and dietitian consultation and support	Outpatient clinic visits at Parkwood Institute for adult (“post-transition”) patients System navigation Telephone/telehealth support for patients and community partners (e.g., Family Physicians, home-care providers) Access to interdisciplinary services, including (but not limited to) physiotherapy, occupational therapy, speech, and language therapy assessment and treatment, and dietitian consultation and support

A physician referral is required to be admitted to the TLC program. Since the inception of the TLC program, patients can be admitted through one of two “pathways”: (1) transition from paediatric care or (2) direct referral from primary care physician after a period of receiving non-specialised care. Patients admitted to the TLC program following a structured transition from paediatric care may transition at any age (up to 21 years) depending on their readiness to transition and where the team feels the patients' needs are best met. Patients admitted to the TLC program from a community primary care physician are often older and have experienced a period where non-specialised/coordinated care was provided only by their primary care physician. After referral, in either pathway, prospective patients are scheduled for an appointment with the physiatrist and/or nurse practitioner, who may refer the patients to other team members for discipline-specific assessment and treatment as appropriate.

### Data Collection

Particular data elements that were extracted from patient medical charts are listed in Table 2. All data extraction was conducted electronically using a data extraction tool created in the REDCap research database platform. In this study, neurological level was defined as the lowest level at which sensory or motor function was preserved in a patient, where function was fully intact above that level. As SB lesions are typically not discrete, with some blurring of normal and abnormal function in the zone of the anatomical spinal cord defect (6), neurological levels were broadly grouped as thoracic, upper lumbar, lower lumbar, and sacral. In the event that patients had asymmetrical neurological presentations on the right and left side, the patient was classified as the higher (i.e., worse) of the two neurological levels. Presenting concerns included any health care or socioeconomic matter that the patient or caregiver felt required the attention of the physician or of another TLC team member, or any issue that the physician felt needed attention at the time of presentation to the TLC program. Comorbid conditions that were controlled or stable and not leading to any active concerns (i.e., were not raised as pressing concerns by the patient/caregiver and/or the physician at the initial clinical consult) were not identified as presenting concerns for this study. Presenting concerns (found within the dictated chart notes of the initial clinical consult, in the concluding treatment plan/list of final recommendations) were summarised and extracted in a list format alongside the collection of other data elements using the REDCap data extraction tool. Patients were included in the study if they presented to the TLC clinic between 2014 and 2017 and had a diagnosis of SB.

**Table 2:** Patient factors and concerns extracted.

**Data elements extracted**
Age (in years, at the time of initial consult)
Date of birth
Sex
Individual reporting concerns (communication status)
*Self, other, unknown, or not reported*
Type of SB
*Myelomeningocele, meningocele, occulta, other (normal exam), or not reported*
Neurological level
*Thoracic, high lumbar, low lumbar, sacral, unknown, or not reported*
Ambulatory status (according to the Hoffer Classification of Ambulation for patients with spina bifida or other diagnoses ((49)))
*Community ambulator, household ambulator, or non-ambulator*
Surgical history
Epilepsy history
Current medications and active non-medical treatment
Physical exam results
*Hip flexion contracture, knee flexion contracture, plantar flexion contracture, or scoliosis*
Presenting issues or concerns at initial consult

### Data Analysis

All presenting concerns (for each patient) were extracted from the REDCap database. From the raw data, each unique health concern was named. With the information extracted from patient medical charts, each patient was then coded as either having or not having each named/identified health concern. Individual health concerns were then grouped into broader “concern categories” to help report the most common concerns across the cohort. Patients were coded as having no concerns, at least one concern, or two or more concerns within each concern category. Descriptive statistics were used to summarise the characteristics of the sample using frequencies, percentages, mean, standard deviation, median, range, minimum, and maximum as appropriate. Concern categories and the individual health concerns within each category were reported as frequencies and percentages. To determine the most common concerns, only the proportion of patients with at least one concern was considered. Total number of concerns were reported as mean, median, standard deviation, and range.

## Results

### Patient Characteristics and Total Concerns

A total of 94 patients with SB were seen in the TLC program between 2014 and 2017 (Table 3). The mean age of the patients was 29.04 years *(SD* = 13.8), where the range was 13–77. Age was not normally distributed, the median was 26 years, the mode was 19 years, and few patients were over the age of 60, establishing that the majority of the patients were within younger age cohorts. The median number of concerns reported at initial consultation was 9.0 (*M* = 9.2, *SD* = 3.9), and ranging from a single concern to a maximum of 22 concerns.

**Table 3:** Demographic characteristics.

**Patient characteristic**	**N(94)**	
	***n***	**%**
**Sex**		
Male	28	29.8
Female	66	70.2
**Communication**		
Self	7	7.4
Other	22	23.4
*SDM*	*21*	*95.5*
*Other*	*1*	*4.5*
Not reported	65	69.1
**SB type**		
Myelomeningocele	72	76.6
Occulta	7	7.4
Other	13	13.8
Not reported	2	2.1
**Neurological level**		
Thoracic	25	26.6
High lumbar (L1-L3)	19	20.2
Low lumbar (L4-L5)	35	37.2
Sacral	10	10.6
Normal exam	5	5.3
**Ambulatory status**		
Community ambulator	40	42.6
Household ambulator	7	7.4
Non-ambulator	47	50
*Independent with transfers*	*31*	*66*
*Assistance with transfers*	*2*	*4.3*
*Dependent with transfers*	*14*	*29.8*
**Surgical history**		
Neurosurgery	70	74.5
Bowel or bladder	19	20.2
Orthopaedic	53	56.4
**Epilepsy history**		
Yes	14	14.9
No	77	81.9
Unclear	2	2.1
Unknown	1	1.1
**Medications**		
Antiepileptic	7	7.4
Psychotropic	11	11.7
Tone	4	4.3
Pain	10	10.6
Bowel/GI	22	23.4
Bladder	33	35.1
Other	31	32.9
**Physical exam results**		
Hip flexion contracture	25	26.6
Knee flexion contracture	34	36.2
Plantar flexion contracture	0	0
Scoliosis	50	53.2

*The “Other” subcategory within “SB Type” represented those with lipomyelomeningocele, dermal sinus tract, occulta, or an unknown SB subtype*.

*The “Normal Exam” subcategory within “Neurological Level” represented patients who had an atypical neurological presentation*.

*The “Other Non-orthopaedic” subcategory within “Surgical History” represented other surgical procedures that were not explicitly collected on the data extraction instrument*.

*“Other” medications included calcium, vitamin D, iron, melatonin, Trazodone, Zopiclone, and Scopolamine*.

### Top Concerns

**Figure 1:**
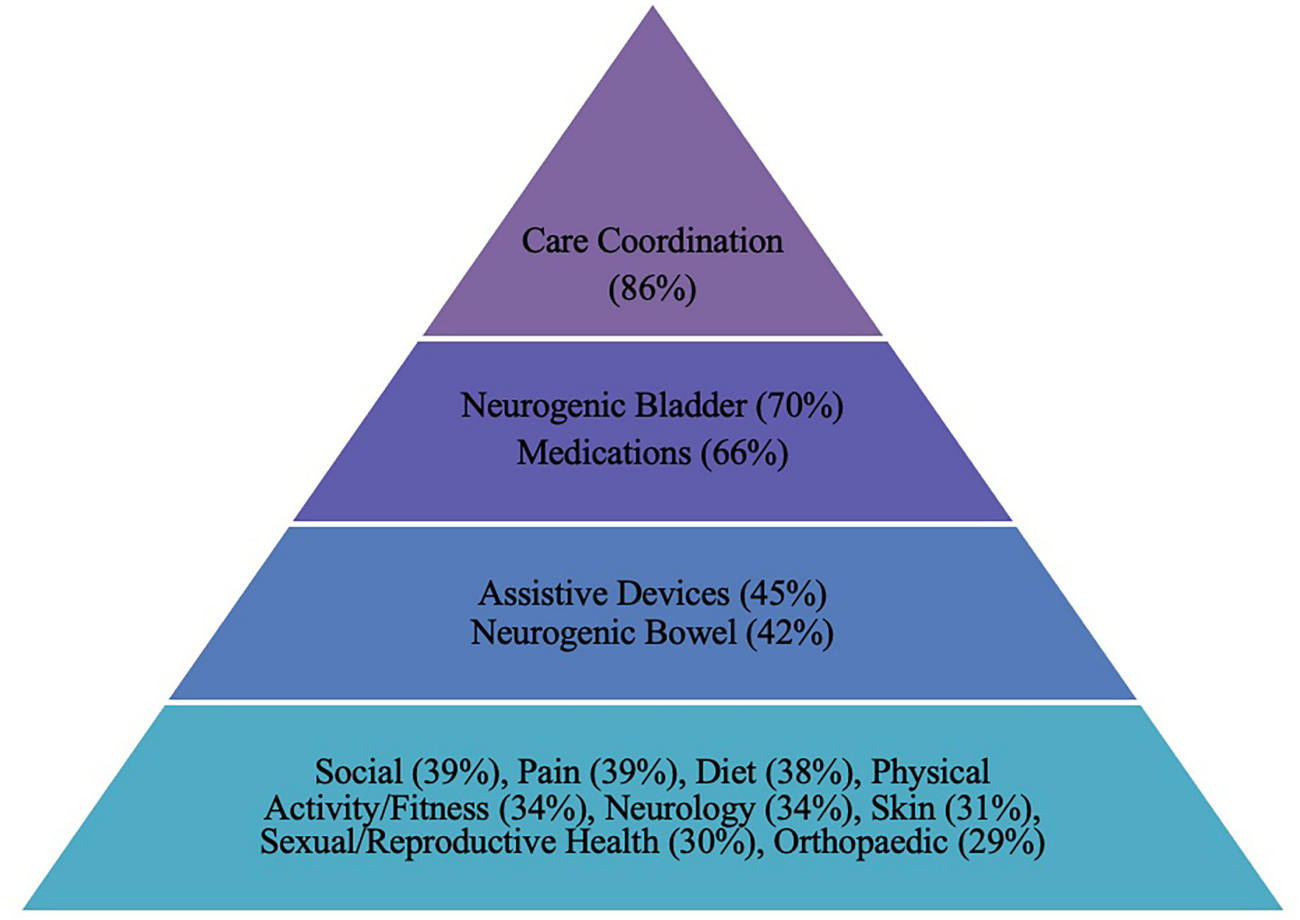
Most common concerns (in order).

One hundred individual patient concerns and 18 concern categories were identified (Supplementary Material). Thirteen health concern categories affected at least 25% of the patient population (Figure 1).

The most common health concern was care coordination (defined as any referral or consideration of another service or specialty within or outside the TLC program), with 86% of patients requiring coordination relating to one or more health issue(s). Unpacking this further, the greatest number of patients required physiotherapy services (35%), most often to address physical activity concerns, and often to address pain, weight loss, wheelchair, and gait concerns. The medical team also referred 15% of patients to recreational therapy, 14% to dietetics, 14% to social work, and 14% to occupational therapy.

Secondly, patients commonly had at least one concern regarding neurogenic bladder (70%), and/or neurogenic bowel (42%). Bladder or kidney concerns included monitoring bladder/renal function (with urinalysis, blood pressure monitoring, renal and bladder ultrasound and/or urodynamics); bladder management (including catheterization, incontinence treatment, hygiene, bladder irrigation, and consideration of need for surgical intervention); bladder education; and concerns about upper or lower urinary tract infections. Health concerns related to neurogenic bowel included constipation, establishing bowel routines, addressing incontinence or changes in bowel patterns, and hygiene.

More than half of the patients (66%) had medication concerns. Medication concerns included requiring or considering new prescriptions; restarting medications; needing to switch or stop medications or alter their dosage; needing to refill existing medications; and concerns with medication compliance. Patient medication concerns were most often related to the management of pain, seizures, bladder or bowel routines, spasticity, hypertension, and ADHD. Several initial clinical encounters also included discussions about supplementation, including vitamin D and calcium for bone health, and magnesium, fibre, or probiotics for bowel management.

Concerns about assistive devices were reported by 45% of patients and mostly involved needs related to orthoses (25%) and wheelchair or seating (19%). Concerns about existing devices, device assessments, and new device options or prescriptions were often discussed.

Social concerns were prevalent (39%) in this population. The most common social concerns related to finances or funding (15%) and social participation (15%). Social participation concerns included an interest in community involvement, opportunities to socialise, leisure activities, support groups, and social programming. Other less common social concerns were associated with school or education (4%) and future care or living arrangements (4%).

In addition, 39% of patients had pain concerns, most commonly back pain (19%), lower extremity pain (14%), and required pain management (14%). Concerns regarding diet were also prevalent, specifically related to food/nutrition (21%) or weight management (13%). A total of 34% of patients had a physical activity or fitness concern. In this area, patients were prescribed specific exercise or physiotherapy programs, stretching, strengthening, and range of motion exercises, and/or had specific concerns about physical activity, exercise, fitness, or sports participation. Furthermore, 34% of patients had concerns about neurologic function, most involving spasticity (12%) and changes possibly related to symptomatic tethered cord syndrome (7%).

Skin health concerns were reported by 31% of the sample and included issues around skin breakdown/redness, the need for plastic surgery, and preventative strategies to maintain skin health. Sexual/reproductive health and family planning concerns were reported by 30% of patients, with family planning being the most common (20%), representing concerns centred around birth control, folate/folic acid supplementation, and SB prevention. Finally, 29% of patients experienced orthopaedic concerns, and 24% had other (miscellaneous) concerns, occurring at too low a frequency to be grouped into concern categories. Some concerns that were less prevalent, but notable, were mental health and functional mobility concerns.

## Discussion

### Top Concerns

In line with the literature (5, 8, 26), patients with SB continue to face multiple conditions associated with SB, affecting multiple organ systems, and creating secondary complications during and after the transition to adult health care. On average, patients had a total of 9 health care concerns and as many as 22 at their initial consultation, with considerable variation in concerns across patients. The results of this study provide evidence that many health concerns persist from childhood and that new health concerns may arise over the life course, necessitating individualised, lifelong care in a medical home, in addition to the support provided during the health care transition from paediatric to adult care.

Care coordination was the most common concern reported by patients with SB, with almost all patients (86%) reporting at least one need in this area. This finding highlights the importance of a service like the TLC program since it can provide access to and coordinate the provision of multidisciplinary care (both within and beyond the program's walls), thus removing the burden of coordinating care from individuals and their families. It also underscores the need for continued access to multiple health care professionals, including allied health, in the management of SB throughout adulthood. Just at the initial clinical encounter, patients were referred to several health care professions, such as physiotherapy, recreation therapy, dietetics, occupational therapy, and social work. In particular, half of the patients had two or more concerns or needs related to accessing and coordinating care among multiple health care services/providers. Many referrals were specifically to services available within the TLC program, where there are knowledgeable clinicians interested in caring for people with complex health care needs. Elsewhere, difficulty with navigating the health care system and accessing competent health care providers who have an interest in SB is common (15). Care coordination and multidisciplinary health services are rarely available for adults with SB (15). Young et al. found that adolescents with SB were more likely to receive health care services from a greater variety of health professionals than adults with SB (27), even though adults with SB continue to identify a wide variety of needs spanning various health care specialties. Many studies have also noted a decline in contact with health and social services after discharge from paediatric care (8, 11, 15). These findings further point (at least partially) to the importance of care coordination and an adult-centred medical home in facilitating access to the appropriate health care professionals in adulthood. The volume of care coordination concerns suggests that the TLC program fills a major gap in the health care system by providing coordinated, multidisciplinary care in a single setting, and serving as a “hub” of knowledge for where and how to access services for patients who are geographically distant.

Previous studies have found that bowel concerns, including faecal incontinence, and bladder concerns, such as incomplete bladder emptying, urinary tract infections, and urinary incontinence, are significant issues for adults with SB (5, 26, 28). The present study further identified a specific need for bladder and kidney monitoring, which is particularly important as renal failure secondary to neurogenic bladder dysfunction continues to be a frequent and sudden cause of death in people with SB (5, 9). As such, bowel and bladder monitoring and management are necessary (1, 4), both at initial consultation and routine follow-up. Additionally, two-thirds of patients had concerns regarding medications, and similar to the findings of Lidal et al. (26), only 10% of participants were not taking any medications at the time of initial consultation. This finding suggests that a medical home plays a significant role in recommending, prescribing, and managing pharmacological treatment and medication regimens for this population (25), which is necessary due to the potential for complications to arise without comprehensive and well-managed treatment strategies (29).

Half of the patients in this sample were non-ambulatory, with only 11% having a sacral neurological level of impairment, pointing at a large proportion of patients with significant limitations in motor function (4). Therefore, it comes as no surprise that concerns relating to assistive devices were prevalent. Assistive devices (such as orthoses, wheelchairs, and gait aids) are commonly used to improve mobility in adults with SB, where preservation of mobility is an important determinant of functioning and quality of life (29). However, many adult practitioners have limited medical training and experience with chronic childhood-onset disabilities (15). The medical home model (in this case, the TLC) mitigates the challenges patients with SB have in accessing services that address their specific assistive device needs within the typical adult care system by providing access to practitioners familiar with the unique and specialised orthotic and device needs of this population. Transition programming must continue to address concerns with existing patient devices and prescribe or advocate for appropriate devices that support mobility, functioning, and participation (29). Physiatrists should also aim to appropriately preserve the physical function and mobility of both ambulatory and non-ambulatory patients through specific rehabilitative programming (29).

Several prominent but less commonly cited concerns identified by this study also warrant clinical consideration, including social concerns, pain, diet, and physical activity. Social well-being is necessary for overall health (30). Many people with SB experience brain abnormalities that present cognitive and physical challenges that can affect independence, education, employment, and/or other social outcomes (4, 31). Research suggests that such consequences may be mitigated or well-managed in a multidisciplinary care setting (31), such as the TLC program. The prevalence of social concerns found in this study suggests that patients experienced challenges in accessing social services, including social work and recreational therapy before the inception of the TLC clinic.

In a recent study conducted by Lidal et al. (26) a cohort of older adults (mean age 58) with SB reported that their most notable health concern was pain. In the current study, while pain was not the most common concern, it affected 40% of patients, with most reporting pain in the lower extremities or the back, in line with the findings in Lidal et al. (26). The younger average age in this study may explain why pain was reported less as, in the literature, pain is more common among older adults with SB (32). A significant portion of patients in the current study also had two or more pain concerns, suggesting that pain in one area may coincide with pain in another body region. The findings from this study further support the literature that adults with SB are likely to experience pain and have a higher prevalence of pain than the general population (33). For this reason, and because pain can affect quality of life and point to underlying treatable conditions (4), specific attention to pain in health care interactions is warranted (4, 26, 34).

Diet is also an important concern to consider in the clinical encounter as a high proportion of older adults with SB are overweight or obese and have hypertension (8, 26). The likelihood of being overweight or obese in adults with SB also increases with age (35) and has been linked to a decline in mobility or ambulatory functioning (26, 35). Another commonly discussed concern at the initial clinical encounter was physical activity/fitness (34%). Physical activity or exercise is particularly important in youth and adults with SB due to their increased risk of obesity, pain, hypertension, and decline in mobility later in life (26). A narrative review of the literature found that adults with SB are more likely to be inactive, have decreased aerobic capacity, lower daily physical activity, and higher levels of obesity compared to other groups of people (36). Bloeman et al. (37) found that children and adolescents who use a manual wheelchair are more sedentary and less physically active than their peers with typical development, increasing the risk for secondary health conditions (37). Exercise training in people with SB can improve elements of fitness (38); therefore, it may be beneficial to consider physical activity as a preventative strategy or to encourage general physical activity for all patients with SB to promote optimal health and well-being. Diet counselling combined with physical activity and fitness programming should be considered essential programming for adults with SB to ensure optimal health and to aid in the prevention of secondary conditions (38, 39). There may be opportunities to run group programming related to dietary and physical activity needs across multiple populations as a cost-effective method of addressing these patient concerns in the current adult healthcare model.

Sexual/reproductive health and family planning (although only a concern for 30% of the population) is also an important area to note considering the younger mean age of this population, the potential for sexual dysfunction in males (4), and the larger number of female SB patients within the TLC clinic who are likely to have unique concerns regarding their reproductive health (puberty, sexuality, pregnancy, childbirth, and menopause) (40). A recent study by Akre et al. (41) also reported that adolescents and young adults with SB report concerns, challenges, and questions regarding their sexuality, fertility, and romantic relationships (41). They reported difficulty in finding answers to their questions and expressed a desire for information directly from their physicians (41). Adult health care providers within the general adult health care system often lack the knowledge and experience to effectively care for people with SB and can be uncomfortable or ambivalent when discussing specific health care topics (15). Meanwhile, health care providers within the TLC program are knowledgeable and interested in caring for the specific needs of this population. The smaller percentage of concerns in this area in this study may reflect that sexual and reproductive health concerns discussed more heavily in follow-up visits once patients feel more comfortable and have established a relationship with the TLC program team. Even so, an opportunity exists to direct more attention to empower and educate youth and adults about their sexual health to further healthy physical and psychosocial development and successful transition to adult care (41).

Two concerns that fell below the threshold set for determining the most “common” concerns (25%) but are worth noting due to the clinical implications include bone health and mental health. The lack of bone health concerns among this cohort may actually reflect suboptimal screening in the initial clinical appointment and point to the “silent” threat of poor bone health, whereby only fractures or significant complications may bring attention to any bone-related issues. On the other hand, since a significant portion of patients were taking vitamin D at the time of consultation, it is also plausible that osteoporosis or fracture risk was considered well-managed among patients transitioning into the program during this period and therefore not identified as a concern. Osteoporosis and bone health are common issues among people with SB and more common in adults with SB than in the general population (5, 42). People with a higher level of neurological impairment and youth are especially at an increased risk of fractures (potentially due to inexperience navigating the physical environment and changes in bone mineral density during adolescence) (43, 44). As such, transition and ongoing adult health care programming must consider bone health in treating and rehabilitating patients with SB and specifically consider risk factors for fractures and low bone mineral density in future clinical encounters (42, 44, 45).

Similarly, mental health concerns were less frequently reported among this group compared to other concerns. Only 15% of the cohort had a specific concern relating to affective disorders, and only a small number of other mental health issues were discussed in the initial consultation. However, mental health problems are frequent and undertreated among people with SB (5, 46). Like sexual and reproductive health concerns, due to the stigma associated with mental illness, patients may prefer to develop a more robust therapeutic relationship before disclosing mental health concerns, where analysis of concerns beyond the initial consultation may have resulted in identifying a higher number of mental health concerns. In a recent study by Dicianno et al. (47), over 25% of adult participants (including younger and older adults) had depressive symptoms, which was comparable to rates in younger people with SB. The authors also estimated that the rate of participants with a history of depression could have been as high as 46% (47). Furthermore, The Mental Health Guidelines for the Care of People with Spina Bifida (46) summarise that due to the social, cognitive, physical, and neuropsychological challenges surrounding SB, people with SB are at risk for symptoms of depression, anxiety, and lower quality of life than the general population (46). Although healthcare providers within the TLC program can screen, provide interventions, and refer patients to the appropriate mental health professionals following the initial patient consultation, specific, concrete strategies should be integrated into all initial clinic visits and follow-up appointments to meet the guidelines proposed by Kritikos et al. (46).

### Considerations

SB is a unique population, and only recently has research begun to explore the specific transition and lifelong care needs of this population. SB can cause complex constellations of clinical features and may present a transition challenge for healthcare providers (16). Adolescents and adults must manage the transfer of multiple areas of care to multiple new health care and social support providers, all while learning to self-manage their condition and comorbidities, advocate for themselves, and coordinate these diverse health care services (16). This transition proves to be even more complex for those who have reduced cognitive functioning and are heavily reliant on caregivers (16). While SB presents unique challenges and clinical manifestations, people with chronic childhood-onset disabilities share a common need for planned and coordinated transition from paediatric to adult health care and ongoing rehabilitative and medical care and social supports throughout adulthood (15, 48).

Interestingly, in evaluating health and health care utilisation among adults with SB from a multidisciplinary adult clinic in the United States, Liptak et al. (8) found that many participants struggled with accessing health care due to inadequate medical resources or for other unspecified reasons. Although programs such as the TLC program may be helpful, it is possible that barriers to accessing care still exist, which may continue to adversely affect health outcomes in adults with SB (37). While program development is still in its infancy, research on optimal processes and outcomes of transition programming is critical to understand what is needed in transition programs to achieve optimal health through adolescence and adulthood for those with childhood-onset disabilities.

As this was a retrospective study evaluating patient needs at the initial clinical encounter with the TLC program, this study only reflects patient concerns at the initial point of contact, during the transition or re-initiation of care with the TLC clinic. Future research will need to determine whether these patient needs have been addressed and if patients are satisfied with the services provided by the TLC program. Additionally, since this study could not address the third aim of the IHI Triple Aim Framework (reduce per capita costs of health care) (24), future research should include economic evaluations of transition programs and medical homes for patients with SB. Future research should also consider mixed-methods or qualitative study designs to fully capture patient and family needs, experiences, and voices.

## Limitations

The major limitation of this study was its retrospective nature. Due to the study design, specific data elements not consistently reported within patient medical charts resulted in difficulties with the standardisation of data collection and extraction of certain data elements. This challenge inevitably led to high percentages of unreported variables of interest, including hip status, that ultimately had to be excluded from this initial study. The concerns identified in this study were derived from clinical consultation notes, which are subject to bias based on how the healthcare provider asked patients and their caregivers about their concerns, and how their concerns were recorded. There could be variability in reporting between providers, including medical residents. Of particular note, there was no method to distinguish between concerns reported by the patient or caregiver and concerns raised by the healthcare provider. Furthermore, the TLC program primarily involves screening by a generalist (physiatrist/nurse practitioner) and referral to other specialists as needed. This program structure is partially responsible for the highest concern category (care coordination) and may have led to some specific concerns not being well-screened for (i.e., mental health concerns, surgical needs, etc.). The wide range in total number of concerns at initial consultation may be partially attributed to the amount of time spent in the clinic visit or the physician's persistence in discussing patient concerns.

This study also has an inherent selection bias in including only TLC patients. Those with less pressing concerns may never have been referred for comprehensive management, thus introducing the possibility of over-estimating the health care needs of this population. Lastly, as age was not normally distributed in this sample, the concerns identified in this study may be more applicable to younger adults (18–21 years of age) and may not reflect the top concerns of adults with SB more generally.

## Conclusion

This study identified many common health concerns in adolescents and adults with SB initiating care in the TLC program. In particular, it established the significance of the usefulness of a medical home in care coordination, medication management, and facilitating assistive device use. It also identified common SB-related health conditions of concern, most notably, neurogenic bladder/bowel. Furthermore, at the initial clinical consultation, patients frequently presented with multiple health concerns. As such, there is a need for continuous and individualised care for people with SB throughout their lifespan, in line with expert recommendations (48). Health care providers within the TLC clinic, and other transition programs and medical homes, must continue to address well-documented concerns, including bladder and bowel, mobility, social, and pain concerns. Consideration must also be given to less frequently discussed topics (identified in the literature as significant in this population), such as sexual health, mental health, and bone health. This research is the first critical step in improving the experience of care and the health of adults with SB through comprehensive and coordinated services. The findings of this study also help to inform priority setting for future development of other transition efforts to improve the standard of care for this population more globally.

## Data Availability Statement

The raw data supporting the conclusions of this article will be made available by the authors, without undue reservation.

## Ethics Statement

The studies involving human participants were reviewed and approved by Western University, the Office of Human Research Ethics. Written informed consent from the participants' legal guardian/next of kin was not required to participate in this study in accordance with the national legislation and the institutional requirements.

## Author Contributions

CC and LB conceptualised, designed, and initiated the study. JS collected the data, performed the analysis, and drafted the manuscript. CC and LB supported data analysis and provided critical feedback to the manuscript. JS, CC, and LB edited the manuscript. All authors contributed to the article and approved the submitted version.

## Funding

Health Care Concerns of Adolescents and Young Adults with Childhood Onset Disabilities. Academic Medical Organization of Southwestern Ontario (AMOSO) Opportunities Fund 2017/9–2020/8.

## Conflict of Interest

The authors declare that the research was conducted in the absence of any commercial or financial relationships that could be construed as a potential conflict of interest.

## Publisher's Note

All claims expressed in this article are solely those of the authors and do not necessarily represent those of their affiliated organizations, or those of the publisher, the editors and the reviewers. Any product that may be evaluated in this article, or claim that may be made by its manufacturer, is not guaranteed or endorsed by the publisher.

## Abbreviations

SB: Spina Bifida
TLC: Transitional and Lifelong Care.
