# Supplementary material for: Health Concerns of Adolescents and Adults With Spina Bifida
Source: Front Neurol. 2021 Nov 12;12:745814. doi: 10.3389/fneur.2021.745814 (PMC8633437; doi:10.3389/fneur.2021.745814)
Supplement: Supplementary file 1 [file Table_1.docx]

Supplementary Material

Table 1. Health Concern Categories and Individual Concerns.

| Concern Category & Individual Concerns | Summary | N | % |
| --- | --- | --- | --- |
| Functional Mobility |  |  |  |
| Improve Ambulation |  | 2 | 2.1 |
| Maintain/Improve Functional Mobility |  | 6 | 6.4 |
| Maintain/Improve Range of Motion |  | 4 | 4.3 |
| Functional Decline |  | 6 | 6.4 |
| Gait |  | 5 | 5.3 |
| Falls/Fall Prevention |  | 3 | 3.2 |
| Functional Mobility Other |  | 2 | 2.1 |
|  | *No concerns* | *73* | *77.7* |
|  | *One concern* | *16* | *17* |
|  | *Two Concerns* | *5* | *5.3* |
| Assistive Devices |  |  |  |
| Orthotics and Braces |  | 23 | 24.5 |
| Wheelchair/Seating |  | 18 | 19.1 |
| Gait Aids |  | 6 | 6.4 |
|  | *No concerns* | *52* | *55.3* |
|  | *One concern* | *37* | *39.4* |
|  | *Two Concerns* | *5* | *5.3* |
| Miscellaneous |  |  |  |
| Swallowing |  | 5 | 5.3 |
| Respiratory |  | 2 | 2.1 |
| Endocrinopathies |  | 2 | 2.1 |
| Inpatient Stay (Care Coordination) |  | 2 | 2.1 |
| Twitching/Cramping |  | 2 | 2.1 |
| Driving |  | 8 | 8.5 |
| Miscellaneous Other |  | 7 | 7.4 |
|  | *No concerns* | *71* | *75.5* |
|  | *One concern* | *19* | *20.2* |
|  | *Two Concerns* | *4* | *4.3* |
| Medications |  |  |  |
|  |  | 62 | 66 |
| Physical Activity/Fitness |  |  |  |
|  |  | 32 | 34 |
| Neurogenic Bladder |  |  |  |
| Current Bladder/Kidney Status/Function |  | 49 | 52.1 |
| Monitoring Bladder/Kidney Health |  | 49 | 52.1 |
|  | *No concerns* | *28* | *29.8* |
|  | *One concern* | *34* | *36.2* |
|  | *Two Concerns* | *32* | *34* |
| Bone Health |  |  |  |
| Bones Health/Fractures |  | 6 | 6.4 |
| Osteoporosis |  | 6 | 6.4 |
| Osteoporosis/Bone Health Treatment |  | 11 | 11.7 |
|  | *No concerns* | *79* | *84* |
|  | *One concern* | *7* | *7.4* |
|  | *Two Concerns* | *8* | *8.5* |
| Neurogenic Bowel |  |  |  |
| Neurogenic Bowel |  | 38 | 40.4 |
| Specific Device Concerns |  | 2 | 2.1 |
|  | *No concerns* | *55* | *58.5* |
|  | *One concern* | *38* | *40.4* |
|  | *Two Concerns* | *1* | *1.1* |
| Care Coordination |  |  |  |
| Physiotherapy |  | 33 | 35.1 |
| Occupational Therapy |  | 13 | 13.8 |
| Rehabilitation Therapy |  | 8 | 8.5 |
| Recreational Therapy |  | 14 | 14.9 |
| Orthotics |  | 7 | 7.4 |
| Dietetics |  | 13 | 13.8 |
| Specialist |  | 8 | 8.5 |
| Family Physician Nurse Practitioner |  | 6 | 6.4 |
| Nephrology |  | 4 | 4.3 |
| Gynaecology |  | 2 | 2.1 |
| Urology |  | 6 | 6.4 |
| Social Work |  | 13 | 13.8 |
| Speech Language Pathology |  | 6 | 6.4 |
| Optometry |  | 2 | 2.1 |
| Family Physician Search |  | 3 | 3.2 |
| Transition/Ongoing Care |  | 11 | 11.7 |
| Seating |  | 11 | 11.7 |
| Wound Clinic |  | 7 | 7.4 |
| Care Coordination Other |  | 4 | 4.3 |
|  | *No concerns* | *13* | *13.8* |
|  | *One concern* | *34* | *36.2* |
|  | *Two Concerns* | *47* | *50* |
| Skin Health |  |  |  |
| Edema Management |  | 4 | 4.3 |
| Pressure Management |  | 10 | 10.6 |
| Wound/Skin Management |  | 20 | 21.3 |
| Edema/Swelling/Lymphedema |  | 4 | 4.3 |
|  | *No concerns* | *65* | *69.1* |
|  | *One concern* | *21* | *22.3* |
|  | *Two Concerns* | *8* | *8.5* |
| Environment |  |  |  |
| Home Accessibility |  | 10 | 10.6 |
| Workplace Accommodations |  | 2 | 2.1 |
|  | *No concerns* | *82* | *87.2* |
|  | *One concern* | *12* | *12.8* |
|  | *Two Concerns* | *0* | *0* |
| Diet |  |  |  |
| Food/Nutrition |  | 20 | 21.3 |
| Weight Management |  | 12 | 12.8 |
| Supplements |  | 4 | 4.3 |
| Dietary Management of Bowels |  | 11 | 11.7 |
|  | *No concerns* | *58* | *61.7* |
|  | *One concern* | *25* | *26.6* |
|  | *Two Concerns* | *11* | *11.7* |
| Sexual/Reproductive Health & Family Planning |  |  |  |
| Women’s Health Issues |  | 4 | 4.3 |
| Family Planning |  | 19 | 20.2 |
| Pregnancy/Childbirth/Childbearing |  | 5 | 5.3 |
| Sexual Health |  | 2 | 2.1 |
| Fertility |  | 6 | 6.4 |
| Menstruation/Menorrhagia |  | 2 | 2.1 |
| Sexual Function |  | 4 | 4.3 |
|  | *No concerns* | *66* | *70.2* |
|  | *One concern* | *16* | *17* |
|  | *Two Concerns* | *12* | *12.8* |
| Pain |  |  |  |
| Lower Extremity Pain |  | 13 | 13.8 |
| Back Pain |  | 18 | 19.1 |
| Upper Extremity Pain |  | 5 | 5.3 |
| Musculoskeletal Pain |  | 2 | 2.1 |
| Pain Management |  | 13 | 13.8 |
| Headaches |  | 3 | 3.2 |
| Bursitis |  | 2 | 2.1 |
| Pain Sensitization |  | 2 | 2.1 |
| Pain Other |  | 3 | 3.2 |
|  | *No concerns* | *57* | *60.6* |
|  | *One concern* | *18* | *19.1* |
|  | *Two Concerns* | *19* | *20.2* |
| Orthopaedic |  |  |  |
| Joint Management |  | 8 | 8.5 |
| Joint Stability/Instability |  | 3 | 3.2 |
| Leg Length Discrepancy |  | 3 | 3.2 |
| Contractures/Flexion Contractures |  | 3 | 3.2 |
| Foot Concerns |  | 4 | 4.3 |
| Spinal Curvature |  | 10 | 10.6 |
|  | *No concerns* | *67* | *71.3* |
|  | *One concern* | *23* | *24.5* |
|  | *Two Concerns* | *4* | *4.3* |
| Mental Health |  |  |  |
| Affective Disorders |  | 14 | 14.9 |
| Coping |  | 2 | 2.1 |
| Behaviour |  | 3 | 3.2 |
| Sleep |  | 3 | 3.2 |
| Substance Use Cessation |  | 3 | 3.2 |
| Mental Health Other |  | 2 | 2.1 |
|  | *No concerns* | *72* | *76.6* |
|  | *One concern* | *17* | *18.1* |
|  | *Two Concerns* | *5* | *5.3* |
| Neurology |  |  |  |
| Neurological Monitoring |  | 2 | 2.1 |
| Numbness/Sensory Loss |  | 2 | 2.1 |
| Vision |  | 3 | 3.2 |
| Articulation/Speech Clarity |  | 2 | 2.1 |
| Seizures |  | 5 | 5.3 |
| Shunt Function |  | 4 | 4.3 |
| Spasticity |  | 11 | 11.7 |
| Symptomatic Tethered Cord |  | 7 | 7.4 |
| Hand Paresthesias/Carpal Tunnel |  | 2 | 2.1 |
| Neurology Other |  | 2 | 2.1 |
|  | *No concerns* | *62* | *66* |
|  | *One concern* | *25* | *26.6* |
|  | *Two Concerns* | *7* | *7.4* |
| Social |  |  |  |
| Return to School |  | 4 | 4.3 |
| Social Support/Participation |  | 14 | 14.9 |
| Independence |  | 5 | 5.3 |
| Employment/Volunteering |  | 6 | 6.4 |
| Future Care/Living Planning |  | 4 | 4.3 |
| Advocacy |  | 2 | 2.1 |
| Transportation |  | 3 | 3.2 |
| Financial/Funding |  | 14 | 14.9 |
| Social Other |  | 3 | 3.2 |
|  | *No concerns* | *57* | *60.6* |
|  | *One concern* | *23* | *24.5* |
|  | *Two Concerns* | *14* | *14.9* |

*Note.* The concerns labelled “__ Other” within each category included individual concerns indicated by only one patient that were therefore grouped together.
